# Associations between ambient air pollution, obesity, and serum vitamin D status in the general population of Korean adults

**DOI:** 10.1186/s12889-022-14164-y

**Published:** 2022-09-17

**Authors:** Byungmi Kim, Juyeon Hwang, Hyejin Lee, Gyeong Min Chae, Seyoung Kim, Hyo-Seon Kim, Bohyun Park, Hyun-Jin Kim

**Affiliations:** 1 National Cancer Control Institute, National Cancer Center, Goyang, 10408 Korea; 2 Cancer Big Data Center, National Cancer Control Institute, National Cancer Center, 323 Ilsan-ro, Ilsandong-gu, Goyang-si, Gyeonggi-do 10408 South Korea

**Keywords:** Ambient air pollution, Chronic exposure, Vitamin D status, General adults

## Abstract

**Background:**

Although a growing body of evidence suggests air pollution is associated with low serum vitamin D status, few studies have reported whether obesity status affects this relationship. The aim of this study was to identify associations between ambient air pollution exposure, obesity, and serum vitamin D status in the general population of South Korea.

**Methods:**

This study was conducted in a cross-sectional design. A total of 30,242 Korean adults from a nationwide general population survey were included for our final analysis. Air pollutants included particulate matter with an aerodynamic diameter ≤ 10 μm (PM_10_), nitrogen dioxide (NO_2_), and carbon monoxide (CO). We measured serum 25-hydroxyvitamin D concentration to assess vitamin D status for each participant. Multiple linear and logistic regression analyses were performed to identify associations between ambient air pollution and vitamin D status in each subgroup according to body mass index level.

**Results:**

The annual average concentrations of PM_10_, NO_2_, and CO were significantly associated with a lower serum vitamin D concentration and higher risk of vitamin D deficiency. The results show a significant association between serum vitamin D status and PM_10_ exposure in obese subgroup. Based on the gender, females with obesity showed more strong association (negative) between different air pollutants and low serum vitamin D concentration and a higher risk of vitamin D deficiency. However, this pattern was not observed in men.

**Conclusions:**

This study provides the first evidence that women with obesity may be more vulnerable to vitamin D deficiency in the context of persistent exposure to air pollution.

**Supplementary Information:**

The online version contains supplementary material available at 10.1186/s12889-022-14164-y.

## Background

The worldwide prevalence of a low vitamin D level is higher than expected despite abundant sun exposure [1], and its prevalence in the general populations, defined as a 25-hydroxyvitamin D (25(OH)D) level below 20 ng/mL, is about 36% in the United States, 61% in Canada, 92% in Northern Europe, 45–98% in Asia, 31% in Australia, and 56% in New Zealand [2]. Vitamin D deficiency is more common in Korea. Since the 2008 Korean National Health and Nutrition Examination Survey (KNHANES), when the measurement of serum vitamin D began, vitamin D deficiency has been increasing continuously in the Korean population [3]. Its prevalence, defined as a serum 25(OH)D concentration < 50 nmol/L, was 51.8% and 68.2% for men and women, respectively, in 2008, but increased to 75.2% and 82.5% in 2014. This prevalence in the Korean population was relatively high when compared to the prevalence in the United States from 2001 to 2006 (29% in men and 34% in women, respectively), adapting the same cutoff for deficiency [4].

Among the factors that can influence the sunlight-induced synthesis of vitamin D, evidence suggests that by absorbing and scattering solar UVB radiation, environmental aerosol pollutants reduce the effectiveness of sun exposure in stimulating the production of vitamin D in the skin [5]. Prospective and observational studies of populations living in different geographic areas have shown that air pollution constitutes an independent risk factor for vitamin D hypovitaminosis [6]. Cross-sectional studies in Iran [7] and China [8] have reported that air pollution increases the prevalence of low vitamin D level. These authors found a higher prevalence of hypovitaminosis D in women living in more polluted areas compared with less polluted.

Obesity is known to be independently related to air pollution [6, 9, 10]. The prevalence of obesity among Korean adults increased steadily over the past few years, from 29.7% in 2009 to 36.3% in 2019 [11]. Previous studies have reported that exposure to air pollution may increase the risk of perivascular and peribronchial inflammation, and may increase both systemic inflammation and oxidative stress, which are the main links between air pollution and obesity [12–14]. Some evidence suggests that exposure to air pollution leads to changes in blood lipids and lipid metabolism through the effects of systemic inflammation [9, 15]. Macrophages are an important source of proinflammatory cytokines in adipose tissue [16, 17].

Serum vitamin D levels are influenced by obesity [6, 18, 19]. Several possible mechanisms may explain the low vitamin D status during obesity. These possible mechanisms include lower dietary intake of vitamin D, less exposure of skin to sunlight because of less outdoor activity, decreased intestinal absorption after malabsorptive bariatric procedures, or impaired 25-hydroxylation and 1-α hydroxylation in adipose tissue in people with obesity [19, 20].

A recent review of the impact of ambient air pollution on serum vitamin D status and obesity has shown that the association between air pollution and vitamin D status tends to vary by body weight status [6]. However, the effect of ambient air pollution on serum vitamin D status and obesity has yet to be studied. Therefore, the aims of this study were to use the KNHANES nationally representative data to identify possible associations between air pollution, serum 25(OH)D concentration, and obesity in Korean adults, and to identify whether any associations differ in relation to air pollution exposure and serum vitamin D status after stratification by body weight status.

## Methods

### Study population

The study sample for this study was obtained from the KNHANES, which was conducted by the Korean Center for Disease Control and Prevention to assess the health and nutritional status of Koreans. The KNHANES is a nationally representative cross-sectional survey of Koreans that used a multi-step cluster probability design as the sampling strategy. KNHANES was launched in 1998 and has been surveyed for 16 years since then. Of these, since vitamin levels were investigated from 2008 to 2014, only data for 7 years were used in this study. This survey collects a variety of information about demographic and socioeconomic factors, health-related behaviors, biochemical profiles, and clinical outcomes. Because vitamin D levels were investigated only from 2008 to 2014, a total of 61,379 people who participated in this period were considered, and 30,242 of whom met all of the following inclusion criteria were included in the present study [missing n (%) = 31,137 (50.7%)]: (1) adults aged ≥ 20 years; (2) those whose records included information about their residential location for estimation of exposure to ambient air pollution; (3) those whose records included serum 25(OH)D concentration; and (4) those who responded accurately to questions about the variables of interest such as their demographics and health-related behaviors. Here, the total number of participants for whom the information on serum vitamin D concentration was missing was 21,619 (0.35%). As shown in Fig. S1, there was no significant difference in the exposure level of each pollutant or the sampling proportion by region before and after the inclusion criteria (Fig. S1). The KNHANES was approved by the Institutional Review Board of the Korea Centers for Disease Control (IRB No. 1401–047–547), and all participants signed an informed consent form. This study meets the Helsinki Declaration based ethical principles for medical research involving human subjects.

### Measurement of air pollution exposure

The exposure to air pollution was assessed using atmospheric monitoring data measured at about 280 monitoring stations nationwide by the Ministry of the Environment of Korea (https://www.airkorea.or.kr). We obtained the annual average values for air pollutant concentration including particulate matter with an aerodynamic diameter ≤ 10 μm (PM_10_), nitrogen dioxide (NO_2_), sulfur dioxide, and carbon monoxide (CO) for 7 years between January 1, 2008, and December 31, 2014, in each administrative division (seven metropolitan cities and eight provinces except for Jeju island). Because we did not have access to the participants’ exact home address, this study design applied a semi-ecological approach in which all participants living in the same administrative district were assigned equal levels of exposure. Therefore, the exposure levels of air pollutants were matched using the annual average value for the administrative district where each individual resided.

### Measurement of serum 25-hydroxyvitamin D concentration

The method for measuring serum 25(OH)D concentration was described in detail in the reports of other KNHANES studies [3, 21]. Briefly, serum 25(OH)D concentration was measured in blood samples obtained from participants after 8 h of fasting. The samples were processed according to the manual, immediately refrigerated and transported to the central testing laboratory, and analyzed within 24 h of transportation. Serum 25(OH)D concentration was measured using a 1470 Wizard gamma counter (Perkin Elmer, Turku, Finland) and a 25-hydroxyvitamin D 125I RIA kit (DiaSorin Inc., Stillwater, MN, USA). The central testing institute in Seoul, Korea participated in the proficiency testing programs of the Vitamin D External Quality Assessment Scheme (DEQAS). The results indicated less than ± 2.0 standard deviation index (SDI), except the those induced by random error. The traceability test performed with the standard reference material (SRM) 972a developed by the National Institute of Standards and Technology (NIST) showed that the measured value was less than ± 10% except for the low concentration values [3]. A cutoff of < 15 ng/mL for serum vitamin D level was used to identify participants with vitamin D deficiency [22, 23].

### Variables of interest

To examine the associations between exposure to air pollution, obesity, and vitamin D status, we included additional data such as demographic variables, lifestyle behaviors, and anthropometric measurements. Demographic factors including sex, age, education level, household income, and residential area were assessed using a questionnaire. We classified the education level into four categories: less than elementary school, middle school, high school, and college or graduate school. Household income was classified into quartiles to adjust for the possible effect of income, and urban and rural areas were classified according to residential administrative district. Behaviors relevant to health, such as smoking status, alcohol consumption, and physical activity, were evaluated using a structured questionnaire and classified as categorical variables as follows: cigarette smoking status (current, former smoker, or never smoker); alcohol consumption (never, less than once a month, two or three times a month, and more than four times a month); and moderate physical activity (yes or no). Anthropometric data including height and weight were also obtained, and the body mass index (BMI) was calculated by dividing the weight (kg) by the square of height (m^2^). Participants were stratified by BMI level into three groups according to the Asia–Pacific obesity classification for adult Asians as follows: underweight or normal weight (BMI < 23 kg/m^2^), overweight (23 kg/m^2^ ≤ BMI < 25 kg/m^2^), and obesity (BMI ≥ 25 kg/m^2^).

### Statistical analysis

Before performing the analyses, we checked the normality assumption for serum vitamin D level and identified a nonnormal distribution. To fit the test’s normality assumptions, square root transformations were applied to the serum 25(OH)D concentration to approximate the normal distribution. The t test and chi-square test were used to compare characteristics between the vitamin D-deficient and normal groups. Multiple linear regression analysis was performed to identify relationships between ambient air pollution and serum 25(OH)D concentration; the results are presented as beta coefficients (βs) and 95% confidence intervals (CIs) for each air pollutant for vitamin D level. Multiple logistic regression analysis was also used to identify any associations between ambient air pollution variables and the presence of vitamin D deficiency; the results are presented as odds ratios (ORs) and 95% confidence intervals (CIs) for each air pollutant for vitamin D deficiency. The statistical estimates, such as *β* coefficients and ORs, for outcomes were converted to interquartile ranges (IQRs) for each air pollutant (9 μg/m^3^ for PM_10_, 11 ppb for NO_2_, and 0.1 ppm for CO). These results were estimated in crude and adjusted models for both sexes as well as the total sample. Confounding factors such as age, sex, education level, household income, survey period, resident region (urbanity), smoking status, alcohol consumption, moderate physical activity, and BMI were included in the adjusted models. We also performed stratified association analyses according to BMI status. All statistical analyses were performed using SAS version 9.3 (SAS Institute, Cary, NC, USA).

## Results

The study characteristics of the participants stratified by vitamin D-deficient (*n* = 10,990) and normal group (*n* = 19,252) are presented in Table 1. The vitamin D-deficient group was slightly younger (46 years) than the normal group (51 years), and the vitamin D-deficient group had a higher percentage of women. In both groups, more than half had a high school or higher education, with the highest percentage at college or high school. A higher percentage lived in urban areas both groups. The percentage of current or former smokers was slightly higher in the normal group (45.3%) than in the vitamin D-deficient group (35.2%). The vitamin D-deficient group had a higher monthly alcohol intake than the normal group. The percentages of participants with overweight or obesity were higher in the normal group than in the vitamin D-deficient group. The mean values for exposure to air pollutants differed between the two groups, especially for NO_2_.

**Table 1:** Characteristics of the study population according to the presence and absence of vitamin D deficiency

Characteristics	Vitamin D deficiency (25(OH)D level < 15 ng/mL)	Normal vitamin D (25(OH)D level ≥ 15 ng/mL)	*p*-value
	Mean ± SD or n (%)	Mean ± SD or n (%)	
n	10,990 (36.3)	19,252 (63.7)	
Age (years)	46 ± 16	51 ± 16	< 0.0001
Women	7,388 (67.2)	9,784 (50.8)	< 0.0001
Education level			< 0.0001
Less than elementary school	2,277 (20.7)	5,457 (28.4)	
Middle school	999 (9.1)	2,316 (12.0)	
High school	3,992 (36.3)	6,283 (32.6)	
College or graduate school	3,722 (33.9)	5,196 (27.0)	
Residential region			< 0.0001
Urban	8,202 (74.6)	12,199 (63.4)	
Rural	2,788 (25.4)	7,053 (36.6)	
Smoking			< 0.0001
Current smoker	2,234 (20.3)	4,365 (22.7)	
Former smoker	1,635 (14.9)	4,350 (22.6)	
Never	7,121 (64.8)	10,537 (54.7)	
Alcohol consumption (times/month)			< 0.0001
Never	3,244 (29.5)	5,191 (27.0)	
≤ 1	3,572 (32.5)	5,108 (26.5)	
2–3	3,637 (33.1)	7,290 (37.9)	
≥ 4	537 (4.9)	1,663 (8.64)	
Physical activity			< 0.0001
Yes	4,022 (36.6)	8,240 (42.8)	
No	6,968 (63.4)	11,012 (57.2)	
Body mass index (kg/m^2^)	23.5 ± 3.5	23.8 ± 3.2	< 0.0001
Obesity status			< 0.0001
Normal	5,296 (48.2)	8,152 (42.3)	
Overweight	2,473 (22.5)	4,730 (24.6)	
Obesity	3,221 (29.3)	6,370 (33.1)	
Serum vitamin D (25(OH)D) level	11.8 ± 2.2	21.6 ± 5.5	< 0.0001
Air pollutant level, mean (median)			
PM_10_ (μg/m^3^)	50.6 (49)	50.2 (49)	< 0.0001
NO_2_ (ppb)	26.1 (28)	24.3 (23)	< 0.0001
CO (ppm)	0.5 (0.6)	0.5 (0.5)	< 0.0001

*PM*_10 _Particulate matter < 10 μm in diameter, *NO*_2 _Nitrogen dioxide, *CO *Carbon monoxide

We also identified the patterns of vitamin D concentration according to the three exposure groups: low (quartile 1), moderate (quartile 2–3), and high exposure (quartile 4). In general, except for PM_10_ concentration exposure in men, as the exposure concentration to each air pollutant increased, the vitamin concentration gradually decreased (Fig. S2).

Simple and multiple linear regression analyses were performed to identify the association between ambient air pollution and quantitative serum 25(OH)D concentration (Table 2). In the total sample, all ambient air pollutants such as PM_10_, NO_2_, and CO levels were significantly associated with a lower serum vitamin D level in both the crude and adjustment models (all *p* < 0.05). These significant associations were also found in women. In men, PM_10_ exposure was not significantly related to serum vitamin D concentration (*p* = 0.41), but NO_2_ (*p* < 0.0001) and CO (*p* = 0.04) exposure were significantly related. The association between air pollution and the presence of vitamin D deficiency was examined using simple and multiple logistic regression analyses. Ambient air pollutant levels were significantly associated with an increased risk of vitamin D deficiency. In the adjusted model, the ORs (95% CIs) for vitamin D deficiency per each IQR increase in PM_10_, NO_2_, and CO were estimated as 1.09 (1.04, 1.14), 1.47 (1.38, 1.56), and 1.16 (1.12, 1.20), respectively. After stratification by sex, the results were similar in women to those for the total sample. In men, as seen for serum vitamin D level, the association between PM_10_ exposure and vitamin D deficiency was not significant (*p* = 0.89).

**Table 2:** Estimated associations of an increase in the IQR for annual average air pollution exposure and serum vitamin D level or the presence of vitamin D deficiency in the total sample

	**Serum vitamin D (25(OH)D) level**					**Presence of vitamin D deficiency**			
	Crude		Adjusted model		Crude			Adjusted model	
	*β (95% CI)*	*p*-value	*β (95% CI)*	*p*-value	*OR* (95% *CI*)		*p*-value	*OR* (95% *CI*)	*p*-value
**Total** (*n* = 30,242)									
PM_10_ (μg/m^3^)	–0.02 (-0.03, -0.01)	0.02	–0.02 (-0.03, -0.01)	0.004	1.10 (1.06, 1.14)		< 0.0001	1.09 (1.04, 1.14)	< 0.0001
NO_2_ (ppb)	–0.20 (-0.21, -0.19)	< 0.0001	–0.13 (-0.15, -0.11)	< 0.0001	1.59 (1.53, 1.66)		< 0.0001	1.47 (1.38, 1.56)	< 0.0001
CO (ppm)	–0.04 (-0.05, -0.03)	< 0.0001	–0.04 (-0.05, -0.03)	< 0.0001	1.16 (1.12, 1.19)		< 0.0001	1.16 (1.12, 1.20)	< 0.0001
**Women** (*n* = 17,172)									
PM_10_ (μg/m^3^)	–0.04 (-0.06, -0.02)	< 0.0001	–0.04 (-0.06, -0.02)	< 0.0001	1.15 (1.10, 1.21)		< 0.0001	1.15 (1.09, 1.21)	< 0.0001
NO_2_ (ppb)	–0.18 (-0.20, -0.16)	< 0.0001	–0.12 (-0.14, -0.10)	< 0.0001	1.556 (1.48, 1.64)		< 0.0001	1.41 (1.31, 1.53)	< 0.0001
CO (ppm)	–0.06 (-0.07, -0.05)	< 0.0001	–0.06 (-0.07, -0.05)	< 0.0001	1.19 (1.14, 1.24)		< 0.0001	1.18 (1.14, 1.23)	< 0.0001
**Men** (*n* = 13,070)									
PM_10_ (μg/m^3^)	0.002 (0.000, 0.004)	0.049	0.009 (-0.01, 0.03)	0.41	1.02 (0.97, 1.09)		0.42	1.01 (0.94, 1.08)	0.89
NO_2_ (ppb)	–0.23 (-0.25, -0.21)	< 0.0001	–0.14 (-0.18, -0.10)	< 0.0001	1.70 (1.59, 1.82)		< 0.0001	1.56 (1.41, 1.72)	< 0.0001
CO (ppm)	–0.01 (-0.02, 0.01)	0.19	–0.02 (-0.04, -0.004)	0.037	1.12 (1.06, 1.17)		< 0.0001	1.12 (1.06, 1.18)	< 0.0001

The ORs and 95% CIs for each air pollutant were scaled to the IQR for each pollutant: 9 μg/m^3^ for PM_10_, 11 ppb for NO_2_, and 0.1 ppm for CO

The adjusted model was adjusted for demographic variables including age, sex, education level, household income, survey period, residential region (urban vs rural), smoking status, alcohol consumption, moderate physical activity, and body mass index

*IQR* Interquartile range, *SE* Standard error, *OR* Odds ratio, *CI* Confidence interval, *PM*_10_ Particulate matter < 10 μm in diameter, *NO*_*2*_ Nitrogen dioxide, *CO* Carbon monoxide

We investigated the effects of exposure to air pollutants on serum vitamin D concentration in each subgroup according to BMI level, and the stratified results are indicated in Table 3. Interestingly, in the total sample, PM_10_ exposure was significantly associated with a lower serum vitamin D concentration in the group with obesity (*p* = 0.002), but not in the groups with normal weight (*p* = 0.39) and overweight (*p* = 0.17). When stratified by sex, the stronger association in the group with obesity than in the groups with normal weight or overweight was more pronounced in women than in men. For women, the levels of air pollutants, including PM_10_, NO_2_, and CO, were significantly associated with a lower vitamin D level in all BMI subgroups, but the effect sizes (*β*) were the largest in the group with obesity. By contrast, the analyses for men showed a different pattern. NO_2_ and CO exposures were not significantly associated with serum vitamin D level in any BMI subgroup. Although NO_2_ exposure was significantly related to lower serum vitamin D levels in all BMI subgroups, the effect size was lowest in the group with obesity [*β* (95% CI) = –0.09 (–0.15, –0.03); *p* = 0.0002] than in the group with normal weight [*β* (95% CI) = –0.19 (–0.25, –0.13); *p* < 0.0001] or group with overweight [*β* (95% CI) = –0.12 (–0.18, –0.06); *p* < 0.0001].

**Table 3:** Estimated associations of an increase in IQR in annual average air pollution exposure and serum vitamin D level according to obesity status

Sample	Exposure	**Normal** (*n* = 13,448)		**Overweight** (*n* = 7,203)		**Obesity** (*n* = 9,591)	
		*β (95% CI)*	*p*-value	*β (95% CI)*	*p*-value	*β (95% CI)*	*p*-value
**Total**	Crude						
	PM_10_ (μg/m^3^)	–0.02 (-0.04, 0.004)	0.12	–0.02 (-0.04, 0.01)	0.25	–0.01 (-0.04, 0.008)	0.21
	NO_2_ (ppb)	–0.23 (-0.25, -0.21)	< 0.0001	–0.19 (-0.23, -0.15)	< 0.0001	–0.17 (-0.19, -0.15)	< 0.0001
	CO (ppm)	–0.04 (-0.06, -0.02)	< 0.0001	–0.03 (-0.05, -0.01)	0.009	–0.046 (-0.07, -0.03)	< 0.0001
	Adjusted model						
	PM_10_ (μg/m^3^)	–0.009 (-0.03, 0.01)	0.39	–0.02 (-0.05, 0.008)	0.17	–0.04 (-0.06, -0.01)	0.002
	NO_2_ (ppb)	–0.13 (-0.17, -0.09)	< 0.0001	–0.11 (-0.15, -0.07)	< 0.0001	–0.12 (-0.16, -0.08)	< 0.0001
	CO (ppm)	–0.03 (-0.05, -0.01)	< 0.0001	–0.03 (-0.05, -0.01)	0.004	–0.06 (-0.08, -0.04)	< 0.0001
**Women**	Crude						
	PM_10_ (μg/m^3^)	–0.04 (-0.06, -0.02)	0.004	–0.05 (-0.09, -0.01)	0.006	–0.045 (-0.08, -0.01)	0.004
	NO_2_ (ppb)	–0.18 (-0.20, -0.16)	< 0.0001	–0.16 (-0.20, -0.12)	< 0.0001	–0.18 (-0.22, -0.14)	< 0.0001
	CO (ppm)	–0.06 (-0.08, -0.04)	< 0.0001	–0.04 (-0.07, -0.01)	0.008	–0.07 (-0.09, -0.05)	< 0.0001
	Adjusted model						
	PM_10_ (μg/m^3^)	–0.03 (-0.05, -0.01)	0.04	–0.05 (-0.09, -0.01)	0.01	–0.07 (-0.11, -0.03)	< 0.0001
	NO_2_ (ppb)	–0.10 (-0.14, -0.06)	< 0.0001	–0.10 (-0.16, -0.04)	0.001	–0.15 (-0.21, -0.09)	< 0.0001
	CO (ppm)	–0.05 (-0.07, -0.03)	< 0.0001	–0.04 (-0.07, -0.01)	0.01	–0.08 (-0.10, -0.06)	< 0.0001
**Men**	Crude						
	PM_10_ (μg/m^3^)	0.03 (-0.01, 0.07)	0.13	0.02 (-0.02, 0.06)	0.38	0.02 (-0.02, 0.06)	0.35
	NO_2_ (ppb)	–0.27 (-0.31, -0.23)	< 0.0001	–0.22 (-0.26, -0.18)	< 0.0001	–0.18 (-0.22, -0.14)	< 0.0001
	CO (ppm)	0.003 (-0.02, 0.02)	0.83	–0.02 (-0.06, 0.02)	0.18	–0.02 (-0.05, 0.007)	0.14
	Adjusted model						
	PM_10_ (μg/m^3^)	0.02 (-0.02, 0.06)	0.30	0.01 (-0.03, 0.05)	0.55	–0.008 (-0.05, 0.03)	0.66
	NO_2_ (ppb)	–0.19 (-0.25, -0.13)	< 0.0001	–0.12 (-0.18, -0.06)	< 0.0001	–0.09 (-0.15, -0.03)	0.0002
	CO (ppm)	–0.01 (-0.03, 0.01)	0.45	–0.02 (-0.06, 0.02)	0.20	–0.03 (-0.05, 0.0008)	0.06

The odds ratios and 95% confidence intervals for each air pollutant were scaled to the IQR for each pollutant: 9 μg/m^3^ for PM_10_, 11 ppb for NO_2_, and 0.1 ppm for CO

The adjusted model was adjusted for demographic variables including age, sex, education level, household income, survey period, residential region (urban vs rural), smoking status, alcohol consumption, and moderate physical activity

*IQR* Interquartile range, *SE* Standard error, *PM*_*10*_ Particulate matter < 10 μm in diameter, *NO*_*2*_ Nitrogen dioxide, *CO* Carbon monoxide

We also evaluated whether the association between ambient air pollution and vitamin D deficiency differed according to BMI level (Table 4). The pattern of overall results for vitamin D deficiency was similar to that of the quantitative serum vitamin D levels. In the total sample, the association between PM_10_ exposure and vitamin D deficiency was significant only in the group with obesity; there was a 1.16-fold increase in the risk of vitamin D deficiency (95% CI = 1.07, 1.25) for each IQR (9 μg/m^3^) increase in PM_10_ concentration. The associations between exposure to air pollutants and the risk of vitamin D deficiency were strongest in women with obesity than in women with normal weight or overweight. For men, PM_10_ exposure was not significantly associated with vitamin D deficiency in any BMI subgroup (all *p* > 0.05). For other pollutants, such as NO_2_ and CO, the risk of vitamin D deficiency was higher in the groups with normal weight and overweight than in the group with obesity.

**Table 4:** Estimated associations of an increase in IQR in annual average air pollution exposure and presence of vitamin D deficiency according to obesity status

Sample	Exposure	**Normal** (*n* = 13,448)		**Overweight** (*n* = 7,203)		**Obesity** (*n* = 9,591)	
		*OR* (95% *CI*)	*p*-value	*OR* (95% *CI*)	*p*-value	*OR* (95% *CI*)	*p*-value
**Total**	Crude						
	PM_10_ (μg/m^3^)	1.09 (1.03, 1.15)	0.002	1.11 (1.03, 1.19)	0.006	1.11 (1.04, 1.18)	0.002
	NO_2_ (ppb)	1.64 (1.55, 1.74)	< 0.0001	1.58 (1.45, 1.71)	< 0.0001	1.52 (1.41, 1.63)	< 0.0001
	CO (ppm)	1.14 (1.09, 1.20)	< 0.0001	1.16 (1.07, 1.23)	< 0.0001	1.18 (1.12, 1.25)	< 0.0001
	Adjusted model						
	PM_10_ (μg/m^3^)	1.06 (0.99, 1.13)	0.08	1.07 (0.98, 1.17)	0.12	1.16 (1.07, 1.25)	0.0002
	NO_2_ (ppb)	1.49 (1.36, 1.63)	< 0.0001	1.4 (1.24, 1.60)	< 0.0001	1.47 (1.31, 1.65)	< 0.0001
	CO (ppm)	1.13 (1.08, 1.19)	< 0.0001	1.16 (1.08, 1.24)	< 0.0001	1.21 (1.14, 1.29)	< 0.0001
**Women**	Crude						
	PM_10_ (μg/m^3^)	1.12 (1.05, 1.20)	0.0007	1.16 (1.05, 1.28)	0.004	1.21 (1.11, 1.32)	< 0.0001
	NO_2_ (ppb)	1.54 (1.43, 1.66)	< 0.0001	1.44 (1.29, 1.61)	< 0.0001	1.63 (1.48, 1.80)	< 0.0001
	CO (ppm)	1.17 (1.11, 1.24)	< 0.0001	1.14 (1.05, 1.24)	0.003	1.27 (1.18, 1.36)	< 0.0001
	Adjusted model						
	PM_10_ (μg/m^3^)	1.10 (1.02, 1.19)	0.02	1.12 (1.1, 1.26)	0.06	1.27 (1.15, 1.40)	< 0.0001
	NO_2_ (ppb)	1.39 (1.25, 1.56)	< 0.0001	1.25 (1.06, 1.48)	0.009	1.59 (1.36, 1.85)	< 0.0001
	CO (ppm)	1.15 (1.08, 1.22)	< 0.0001	1.13 (1.03, 1.23)	0.010	1.30 (1.20, 1.41)	< 0.0001
**Men**	Crude						
	PM_10_ (μg/m^3^)	1.01 (0.92, 1.11)	0.85	1.07 (0.96, 1.21)	0.22	1.01 (0.92, 1.12)	0.83
	NO_2_ (ppb)	1.77 (1.60, 1.97)	< 0.0001	1.85 (1.63, 2.11)	< 0.0001	1.54 (1.38, 1.72)	< 0.0001
	CO (ppm)	1.09 (1.01, 1.18)	0.03	1.21 (1.10, 1.34)	0.0002	1.09 (0.99, 1.19)	0.062
	Adjusted model						
	PM_10_ (μg/m^3^)	0.98 (0.88, 1.09)	0.73	1.01 (0.88, 1.16)	0.87	1.03 (0.92, 1.16)	0.58
	NO_2_ (ppb)	1.73 (1.47, 2.03)	< 0.0001	1.65 (1.35, 2.01)	< 0.0001	1.33 (1.12, 1.58)	0.001
	CO (ppm)	1.11 (1.02, 1.20)	0.02	1.19 (1.07, 1.33)	0.002	1.09 (0.99, 1.20)	0.09

The ORs and 95% CIs for each air pollutant were scaled to the IQR for each pollutant: 9 μg/m^3^ for PM_10_, 11 ppb for NO_2_, and 0.1 ppm for CO

The adjust model was adjusted for demographic variables including age, sex, education level, household income, survey period, residential region (urban vs rural), smoking status, alcohol consumption, and moderate physical activity

*IQR* Interquartile range, *OR* Odds ratio, *CI* Confidence interval, *PM*_*10*_ Particulate matter < 10 μm in diameter, *NO*_*2*_ Nitrogen dioxide, *CO* Carbon monoxide

These results were stratified into two groups (with and without obesity, and men and women). In women, the association between air pollutants and vitamin D deficiency was stronger in the group with obesity than in the other group, but this pattern was not observed in men (Fig. S3).

## Discussion

The aim of this study was to determine whether exposure to air pollution is related to serum 25(OH)D concentration or obesity in Korean adults. We found inverse associations between exposure to ambient air pollution and serum 25(OH)D concentration or vitamin D deficiency. This study supports the idea of a vicious cycle involving low vitamin D status, exposure to air pollution, and obesity. The detrimental effects seemed to be additive for participants with obesity, especially in women.

The findings of this study are consistent with prior studies showing that people exposed to higher levels of air pollution have lower serum 25(OH)D concentration or are at increased risk of developing vitamin D deficiency. A cross-sectional population-based study in China, India, and Iran found that vitamin D deficiency was related to air pollution [7, 8, 24]. These studies typically used citywide monitoring measurements as a proxy for population exposure instead of assessing individual level personal-exposure to air pollution. Moreover, only one cohort study has investigated the potential effects of exposure to air pollution on circulating serum 25(OH)D concentrations in the general population [25]. This large UK prospective cohort study from 22 assessment centers in England, Wales, and Scotland also observed that long-term exposure to PM_2.5,_ PM_10_, NO_X_, and NO_2_ were associated with lower serum 25(OH)D concentrations. This association between air pollution and lower serum vitamin D level was clearer in women [25]. After adjusting for potential confounding factors, a 10 μg/m^3^ increase in the concentrations of PM_2.5,_ PM_10_, NO_X_, and NO_2_ in women was associated with 14.69 (95% CI: 8.69 to 20.70), 4.25 (95% CI: 1.30 to 7.20), 0.69 (95% CI: 0.32 to 1.06), and 1.94 (95% CI: 1,17 to 2.71) nmol/L decreases in serum 25(OH)D concentrations, respectively. However, the study by Yang et al. found no association between NOx, NO_2_, and serum vitamin D levels in men. Other studies have assessed the effects of air pollution on serum vitamin D level, but the findings have been inconclusive [8, 26–29]. The inconsistency may reflect differences in sample sizes, air pollution metrics, geographic conditions, or assessment of exposure.

Previous epidemiological studies suggest that exposure to pollutants can induce both systemic inflammation and oxidative stress, the main links of air pollution with obesity [12–14]. More recent evidence suggests that obesity influences the relationship between air pollution exposure and changes in the lipid profile [9, 10, 30, 31]. In addition, Scott Weichenthal et al. reviewed the literature and suggested that people with obesity may be most susceptible to the adverse cardiovascular risks of air pollution exposure after adjusting for potential confounding factors [32].

Obesity is strongly related to a low vitamin D level because a higher BMI leads to lower vitamin D level [33]. Vitamin D receptors are widely expressed in adipose and β-pancreatic cells, and both cell types have the enzyme 25-hydroxyvitamin D 1-α-hydroxylase and can therefore activate vitamin D [18]. Through its receptors, vitamin D exerts its effects in vitro on adipocyte lipid metabolism and adipocyte gene expression [6].

Although vitamin D status and exposure to air pollution affect the risk of obesity, previous studies have not investigated the potential effects of exposure to air pollution on serum 25(OH)D concentration in subgroup analysis stratified according to obesity status. Our findings provide the first evidence of significant inverse associations between air pollutions and serum 25(OH)D concentration and vitamin D deficiency in people with obesity. In addition, these associations seem to be stronger in women than in men. Although the evidence to support this is unclear, various risk factors, such as pregnancy and menopause, which are linked to vitamin D concentrations, may influence this association. However, further studies need to be conducted to confirm these associations.

The mechanism by which air pollution decreases serum 25(OH)D concentration in people with obesity is not fully understood. However, a great body of evidence indicates that air pollutants reduce the effectiveness of sun exposure in producing vitamin D in the skin by absorbing and scattering solar UVB radiation [5, 6, 28]. Air pollutions effectively absorbs UVB radiation, thereby reducing the quantity of photons reaching the earth’s surface. According to a recent review of previous studies, the associations among the vitamin D status, air pollution and obesity may provide a rationale for considering obesity as an additional link between air pollution and low vitamin D status [6]. Air pollutants may contribute to the risk of obesity as environmental obesogens through a vicious cycle involving exposure to air pollutants, absorption of solar UVB radiation, and reduction in the effectiveness of sun exposure in inducing vitamin D production in the skin [6]. In addition, it has also been suggested that accrual of adipose tissue obesity could result from an excessive adaptive “winter response”, and that the decline in vitamin D skin synthesis, due to reduced sun exposure, contributes to the tendency to increase fat mass during the cold season [34]. Other studies have reported that exposure to urban air pollution in healthy children is associated with systemic inflammation, endothelial damage changes in the activity of and sensitivity to appetite-regulating peptides, and an increased risk of obesity and vitamin D deficiency [35, 36]. Besides air pollutants, several studies report smoking was significantly associated with low serum vitamin D level [37–39]. The decreased serum 25(OH)D concentrations seen in smokers might be due to accumulation of cadmium in the kidney [37]. However, findings regarding the vitamin D status in smokers are conflicting [38]. The mechanisms behind the finding need further exploration.

To our knowledge, this study is the first to report on the effects of obesity on the association between air pollution and vitamin D status in the general population of Korean adults. Our study has some limitations. First, because of the cross-sectional design of the KNHANES, it was not possible to determine any causal associations between exposure to air pollution, vitamin D status, and obesity. Confirmation from prospective cohort studies is needed to validate our findings. Second, the rate of missing data for serum 25(OH)D concentration in this study was high in 2013–2014, which may indicate a loss of representativeness of the total population. Nevertheless, we extracted individual data for serum 25(OH)D concentrations in the KNHANES 2008–2014 data according to the survey district, sex, and age. This probably ensures the representativeness of our data, although the risk of observation bias may remain. Third, we did not collect information about vitamin D supplementation, and future studies should include this information. Fourth, we did not apply accurate exposure assessments to predict annually averaged exposure by considering variables related to land use, road traffic proxies, topography, climate, and population density. Therefore, we may have over- or underestimated each participant’s exposure to air pollutants. Fifth, various information such as seasonal effect, temperature and solar radiation were not considered as confounding factors, due to the absence of relevant data. Therefore, the results may be likely to be affected by residual bias. Sixth, we did not have access to data on inflammatory biomarkers such as tumor necrosis factor-alpha, Interleukin-10, and C-reactive protein, which could have substantiated our reported association between air pollution and serum 25(OH)D concentrations. Seventh, we could not include ozone measurements in the analyses, which are related to solar UV-B radiation due to concerns about the quality-assessment of ozone examination. It needs to be reconfirmed in future studies. Finally, we did not include data on exposure to PM_2.5_ and exposure to other environmental pollutants including endocrine-disrupting chemicals such as phthalate and bisphenol A, which can also decrease serum 25(OH)D concentration in the general population [40].

## Conclusions

Air pollution was found to be inversely associated with low vitamin D status in the general population in Korea, especially in the population with obesity. We suggest that there may be a vicious cycle between low vitamin D status, exposure to air pollution, and obesity. Further prospective studies are needed to confirm whether the associations between vitamin D levels, air pollutant exposure, and obesity are causal, and the potential for nutritional interventions to reduce the detrimental effects of exposure to air pollutants and to prevent vitamin D deficiency during weight loss in adults.

## Supplementary Information

**Additional file 1:Figure S1.** The exposure level to each air pollutant before and after the inclusion criteria. The color concentration on the map indicates the exposure level of each pollutant. The size of the circle indicates the proportion of participants who participated in the study by administrative district. **Figure S2.** Exposure-response graph between three air pollution exposure levels and vitamin D conc (a) for men and (b) for women. **Figure S3.** Estimated associations of an increase in IQR on annual average air pollution exposure and presence of vitamin D deficiency in data stratified into two groups (groups with and without obesity; men and women).

## Data Availability

The datasets analyzed during the current study are available in website of The Korea Disease Control and Prevention Agency; Korea National Health and Nutrition Examination Survey repository, [https://knhanes.kdca.go.kr/knhanes/sub03/sub03_01.do].
