# Supplementary material for: Associations between ambient air pollution, obesity, and serum vitamin D status in the general population of Korean adults
Source: BMC Public Health. 2022 Sep 17;22:1766. doi: 10.1186/s12889-022-14164-y (PMC9482201; doi:10.1186/s12889-022-14164-y)
Supplement: Supplementary file 1 — Additional file 1:Figure S1. The exposure level to each air pollutant before and after the inclusion criteria. The color concentration on the map indicates the exposure level of each pollutant. The size of the circle indicates the proportion of participants who participated in the study by administrative district. Figure S2. Exposure-response graph between three air pollution exposure levels and vitamin D conc (a) for men and (b) for women. Figure S3. Estimated associations of an increase in IQR on annual average air pollution exposure and presence of vitamin D deficiency in data stratified into two groups (groups with and without obesity; men and women). [file 12889_2022_14164_MOESM1_ESM.docx]

**Supplementary Files**


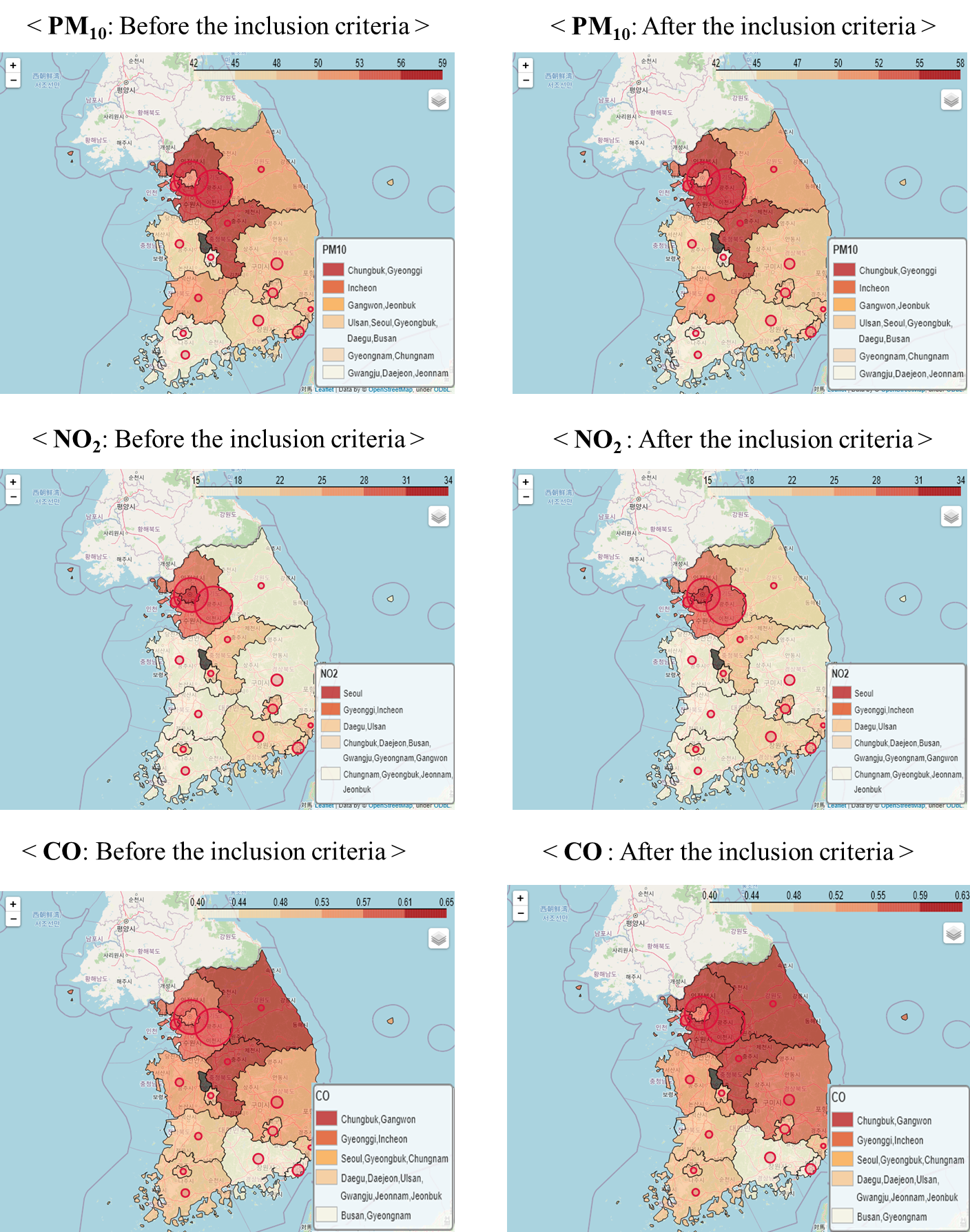


Figure S1. The exposure level to each air pollutant before and after the inclusion criteria. The color concentration on the map indicates the exposure level of each pollutant. The size of the circle indicates the proportion of participants who participated in the study by administrative district.


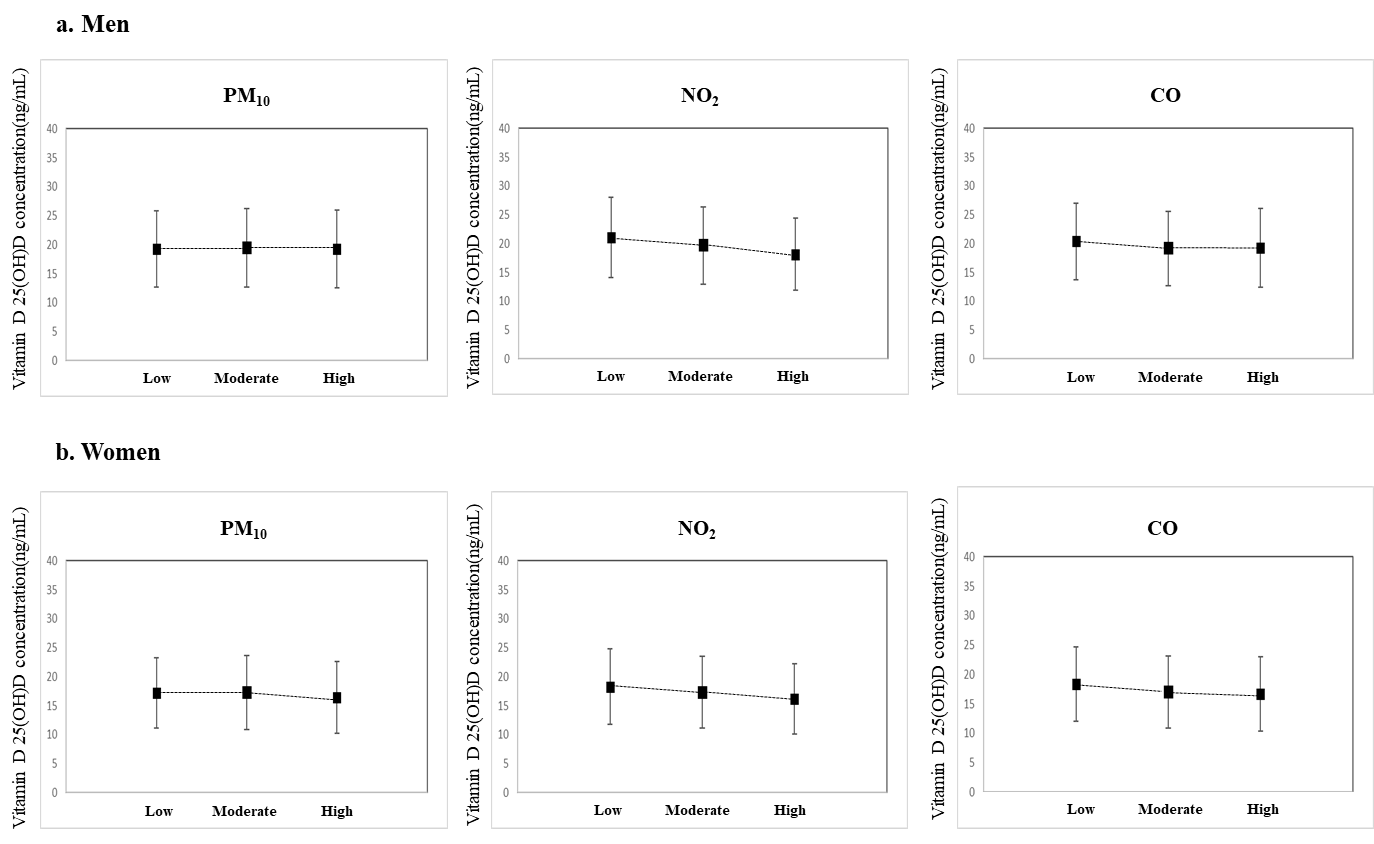


Figure S2. Exposure-response graph between three air pollution exposure levels and vitamin D conc (a) for men and (b) for women.


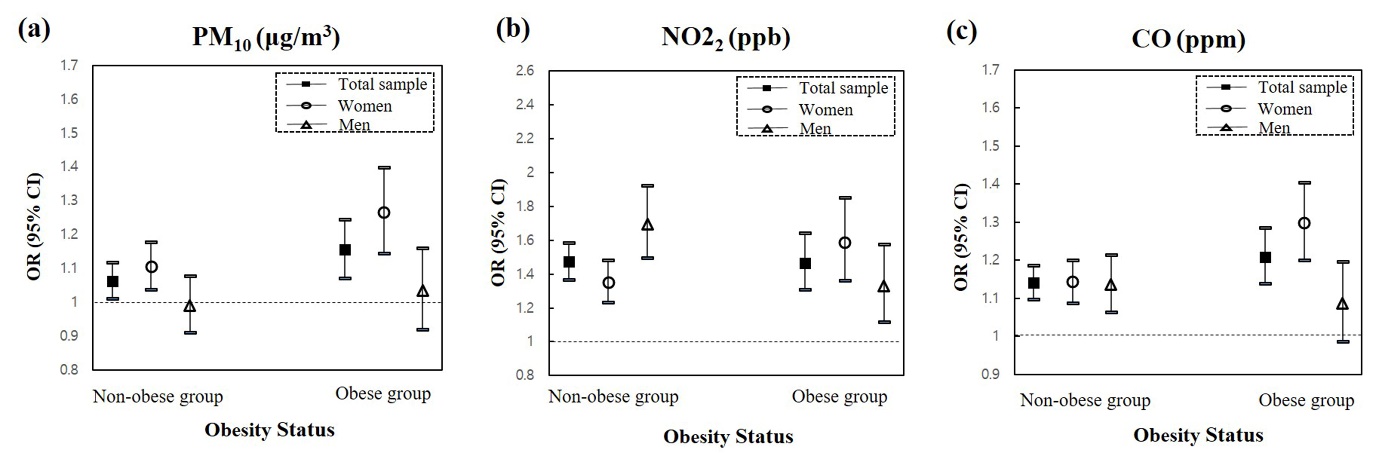


Figure S3. Estimated associations of an increase in IQR on annual average air pollution exposure and presence of vitamin D deficiency in data stratified into two groups (groups with and without obesity; men and women).
